# Clinical utility of self-expandable metal stents in the treatment of anastomotic obstruction secondary to recurrent gastric cancer

**DOI:** 10.3389/fonc.2025.1599582

**Published:** 2025-06-18

**Authors:** Haiyang Lai, Ketong Wu, Yang Liu, Dan Li, Tao Peng, Yuan Wan, Bo Zhang

**Affiliations:** Department of Interventional Center, Biomedical Innovation Center, The Sixth Affiliated Hospital, Sun Yat-sen University, Guangzhou, China

**Keywords:** anastomotic obstruction, self-expandable metal stents, gastric cancer, gastrojejunostomy, esophagojejunostomy

## Abstract

**Aim:**

The purpose of this study was to assess the efficacy and safety of self−expandable metal stents (SEMS) in treating anastomotic obstruction associated with recurrent gastric cancer.

**Methods:**

Ten patients with anastomotic obstruction in recurrent gastric cancer were treated by SEMS implantation under fluoroscopic guidance. All patients presented with refractory nausea, vomiting and complete inability to tolerate oral intake before stent placement, requiring total parenteral nutrition (TPN). Clinical data were retrospectively analyzed the technical and clinical success rates, stent patency and complication rates.

**Results:**

SEMS was successfully implanted in all patients, and clinical success rate was 100%. The operations were subtotal gastrectomy with Billroth-II reconstruction (n = 3), radical distal gastrectomy (n = 3), total gastrectomy with esophagojejunostomy (n = 3), and palliative gastrojejunostomy (n = 1). Three patients developed stent occlusion due to intrastent tumor ingrowth secondary to disease progression after initial anastomotic stent placement, and underwent secondary stent implantation with successful maintenance of patency postoperatively. One patient developed stent obstruction due to food impaction on postoperative day 10, which was managed endoscopically with successful restoration and maintenance of luminal patency. The mean stent patency was 78 d (range, 8–225 d). No serious complications, such as anastomotic leakage, stent migration and bleeding were observed in these patients.

**Conclusions:**

Fluoroscopically-guided SEMS placement represents a technically safe and clinically effective intervention for managing anastomotic obstructions in recurrent gastric cancer. SEMS placement offers rapid symptom relief, shorter hospital stays, and improved quality of life compared to surgical alternatives in this patient population. Thus, based on its technical feasibility and clinical outcomes, this method warrants primary consideration in palliative treatment algorithms.

## Introduction

Gastric cancer is a highly malignant digestive system tumor with a bad prognosis. According to the current global cancer statistics, gastric cancer is one of the top five in both morbidity and mortality rates worldwide (1). Anastomotic obstruction with tumor recurrence occurred in approximately 20% of post-gastrectomy patients (2, 3). Due to the lack of effective therapeutic options, the prognosis remained extremely poor, significantly impairing quality of life and reducing overall survival (4). Cancer recurrence at the surgical junction after gastric cancer resection presents with nonspecific symptoms. The primary manifestations include postprandial nausea, vomiting, and dysphagia, while a minority of patients present with epigastric distension, melena, or hematemesis (5). The priority treatment is providing nutritional supporting and improving their quality of life, such as oral intake and relieving nausea and vomiting (6).

Despite the potential method for symptom relief through surgical management, the associated morbidity and mortality remain substantial, and successful outcomes are limited to approximately half of the treated patients (7). For patients with anastomotic obstruction in recurrent gastric cancer, malnutrition secondary to oral intake failure often precludes repeat surgery (8). Moreover, even after successful reoperation, the mandatory delay in chemotherapy during recovery results in early recurrence, adversely affecting both quality of life and survival outcomes (9).

SEMS placement had been demonstrated as a viable alternative to surgery for managing anastomotic obstruction, particularly in patients with limited life expectancies (10). As a minimally invasive intervention, SEMS offered an effective and safe palliative option for advanced cancer patients with anastomotic obstruction (11, 12). Compared to surgery, stent placement provided distinct advantages, including reduced mortality rates, shorter hospitalization periods, and quicker symptom alleviation (13). Given the context of terminal malignancies, multiple comorbidities, limited survival, or advanced age, SEMS was considered the first-line therapeutic approach for anastomotic obstruction.

Although the effectiveness and safety profile of SEMS had been well-documented in the setting of unresectable gastric cancer complicated by gastric outlet obstruction (GOO), only a small number of studies have examined SEMS for anastomotic obstruction in recurrent gastric cancer, resulting in limited evidence on their efficacy. The purpose of this study was to assess the efficacy and safety of SEMS in treating anastomotic obstruction associated with recurrent gastric cancer.

## Materials and methods

### Patients

All consecutive patients who had inserted SEMS for recurrent gastric cancer-related anastomotic obstruction during the study period (March, 2018–November, 2023) were included. Patient demographics and baseline characteristics were presented in **Table 1**. The patients with obstruction due to anastomotic tumor recurrence, the low diagnostic yield of endoscopy and limited tissue sampling often complicated histopathological confirmation. Therefore, the diagnosis should be established through a combination of contrast-enhanced abdominal CT, upper gastrointestinal contrast and clinical manifestations. Further treatment strategies were developed through multidisciplinary team (MDT) consultations to ensure optimal patient care.

**Table 1:** The clinical data of study cases.

Patient no	Age (y)	TNM stage	Type of surgery	Stenosis site	Length of stenosis (cm)	GOOSS		Patency of SEMS (days)	Follow-up (days)
						Before	After		
1	54/M	T3N0M0	TG	E–J	3.5	0	3	81	Died after 81 days
2	42/M	T4bN3M0	TG	E–J	4.0	0	3	131	Died after 193 days
3	48/M	T3N0M0	Bll	G–J	5.0	0	3	225	Died after 328 days
4	64/M	T4bN3M0	Bll	G–J	3.0	0	2	27	Died after 64 days
5	47/M	T3N1M0	Bll	G–J	2.0	0	3	76	Died after 87 days
6	58/M	T3N2M1c	GJ	G–J	4.5	0	1	8	Died after 67 days
7	59/M	T3N3M0	DG	G–J	3.0	0	1	34	Missing
8	43/F	T4aN1M0	DG	G–J	4.0	0	2	78	Died after 163 days
9	66/M	T3N2M0	TG	E–J	3.5	0	3	101	Died after 107 days
10	58/M	T4N2M0	DG	G–J	2.0	0	2	27	Missing

M, Male; F, female; TG, Total Gastrectomy; BII, Bilroth type II anastomosis; G-J, gastrojejunostomy; DG, Distal Gastrectomy; E-J, esophagojejunostomy; Gastric Outlet Obstruction Scoring System (GOOSS) as follows: 0 = no oral intake; 1 = exclusively liquid diet; 2= exclusively soft solids diet; 3 = full diet possible; SEMS, self-expandable metal stents.

The inclusion criteria were as follows: (1) Upper gastrointestinal contrast imaging revealed dilated fluid-filled bowel loops proximal to the anastomosis, approximate anastomotic occlusion, and failure of contrast agent passage; (2) contrast-enhanced CT or PET/CT imaging confirmed localized thickening with enhancement at the anastomotic site, while endoscopic or laparoscopic examination demonstrated tumor recurrence at the anastomosis. The exclusion criteria comprised distal small bowel or colorectal obstructions that were either multi-segmental or of closed-loop configuration.

Written informed consent was obtained from all patients prior to SEMS placement.

### Procedures

The procedure was initiated with the patient in standard supine position. Using continuous fluoroscopic visualization, we established transnasal access by a 0.90 mm-wide, 150 cm-long guidewire (Terumo Corporation, Tokyo, Japan) and a 5-Fr DAV catheter (Cook Medical, Bloomington, IN, USA) via an 8-Fr introducer sheath (Boston Scientific, Marlborough, MA, USA). The assembly was carefully advanced through the upper digestive tract until reaching the proximal margin of the anastomotic stricture. Following guidewire withdrawal, we performed contrast injection (iodinated agent) through the indwelling catheter to: Precisely localize the stricture; Determine the degree of luminal compromise; Measure the involved segment length. Subsequently, under fluoroscopic monitoring, the guidewire was reintroduced and the catheter system was delicately advanced across the stenotic region using standard interventional techniques. A 450 cm Zebra guidewire (0.035-inch diameter; Boston Scientific, Natick, MA) was then advanced through the catheter lumen, with its tip positioned securely in the proximal jejunum under fluoroscopic guidance. After achieving optimal wire position: The DAV catheter was carefully withdrawn; The introducer sheath was removed; The Zebra guidewire remained *in situ* as a stable access platform. A SEMS (Cook Medical, Limerick, Ireland) of appropriate length was deployed over the guidewire to bridge the stenotic anastomosis. In cases of suboptimal stent expansion, a nasojejunal tube (Wilson-Cook Medical, USA) was inserted through the stent lumen along the pre-placed Zebra guidewire for enteral access. Serial abdominal radiographs were obtained at 48–72 hours intervals to monitor stent deployment and positional integrity. In cases of intraluminal restenosis, duodenal stent placement was performed using a stent-within-stent approach to maintain luminal patency.

### Observation of efficacy

Stent placement was deemed technically successful upon meeting all of the following criteria: Adequate stent deployment across the anastomotic occlusion, with the stent extending at least 2 cm beyond the stenotic segment at both ends. Post-procedural abdominal X-ray demonstrating contrast passage through the stent from the proximally dilated bowel into the distal small intestine, indicating restoration of luminal continuity (14, 15). Clinical success was defined as: Significant symptomatic relief within 1–3 days post-stent placement, including: Tolerance of oral intake (liquid/semisolid diet); Resolution of obstructive symptoms (e.g., vomiting, abdominal distension); Absence of procedure-related complications (e.g., bleeding, perforation, stent migration) during the perioperative period (16, 17). Complications included anastomotic leakage, stent migration and bleeding.

### Statistical analysis

Continuous variables were presented as mean ± standard error of the mean along with their range, while qualitative variables were expressed in terms of absolute and relative frequencies. Stent patency analysis utilized Kaplan-Meier methodology with log-rank testing (SPSS v29.0, IBM Corp). Statistical significance threshold was p<0.05.

## Results

### Clinical characteristics

**Figure 1:**
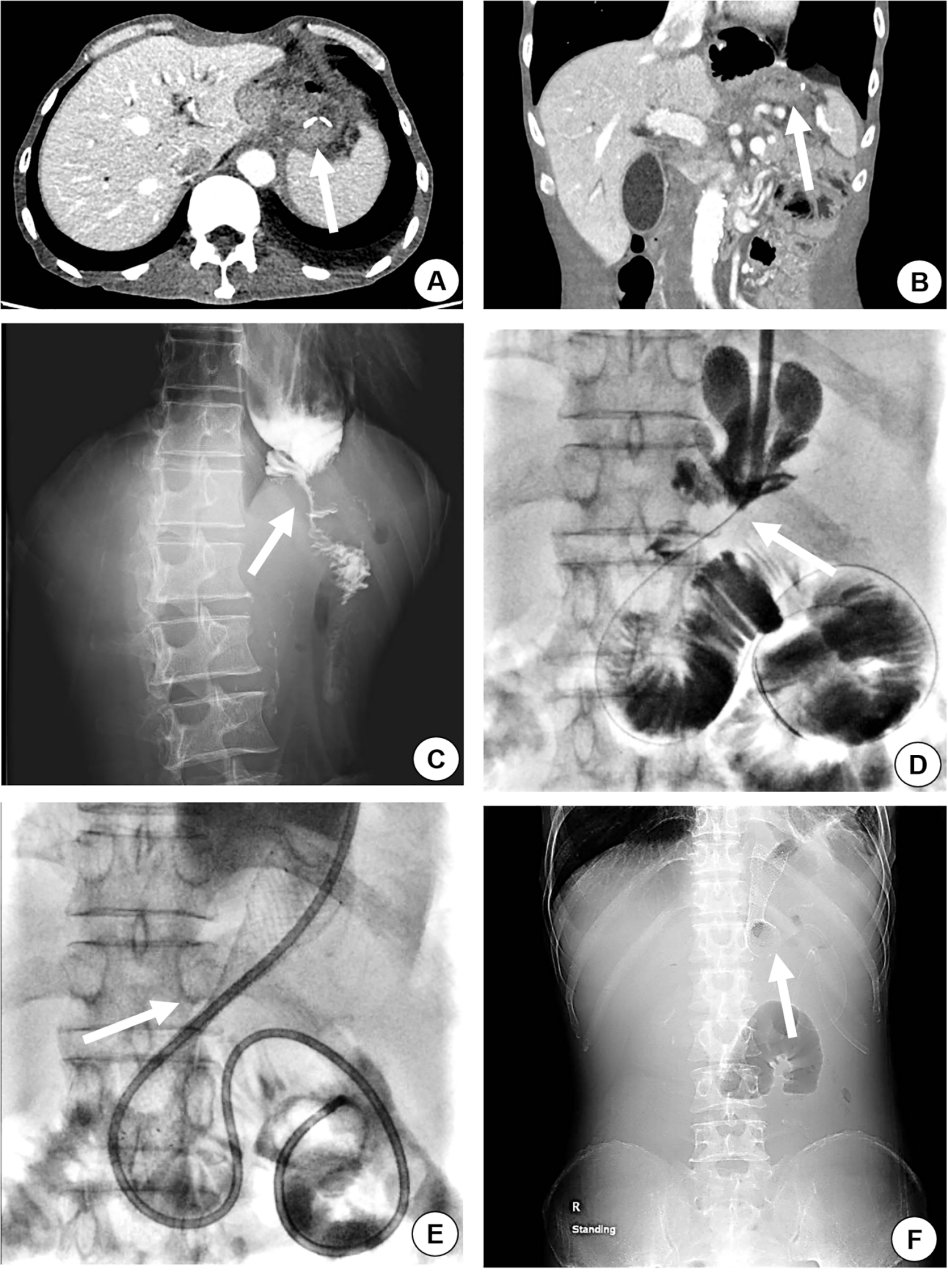
A 54-year-old male, with a history of total gastrectomy performed over 2 years prior, was admitted for recurrent nausea, vomiting, and failure to tolerate oral intake. Abdominal contrast-enhanced CT (axial and coronal planes) demonstrated anastomotic thickening and significant enhancement (arrow), radiologically suspicious for recurrent malignancy **(A, B)**. Upper gastrointestinal contrast study revealed severe stenosis at the anastomotic site, with contrast agent passing through in a thin, thread-like stream (**C**, arrow). The iodinated contrast media was slowly injected to confirm the site of anastomosis stenosis (**D**, arrow), and the stent was placed in occluded segment of the site (**E**, arrow). A plain X-ray radiograph of the abdomen was taken to evaluate the site and dilation of the stent (**F**, arrow).

Our study cohort consisted of 10 patients (9 male, 90%; 1 female, 10%) with a mean age of 53.9 ± 8.1 years (range: 42–66 years). Surgical histories included: Billroth-II reconstruction after subtotal gastrectomy (n = 3), radical distal gastrectomy (n = 3), total gastrectomy with esophagojejunostomy (n = 3), and palliative gastrojejunostomy (n = 1). All patients received bare metal stents (22mm diameter, 9-12cm length). The mean procedure duration was 26.3 ± 12.2 minutes (range 10-50). The mean length of stenosis was 4.05 ± 0.85 cm (range 3.0-6.0). Nutritional recovery was objectively assessed using the Gastric Outlet Obstruction Scoring System (GOOSS). All patients (100%) demonstrated complete symptomatic relief within 72 hours post-intervention (**Figure 1**). Following stent placement, all patients resumed oral intake: 2 patients tolerated a liquid diet (e.g., broth, juice), 3 patients progressed to a soft solid diet (e.g., porridge, mashed potatoes), 5 patients successfully transitioned to nearly all types of regular diet. Seven patients underwent multiple cycles of palliative chemotherapy following stent placement, the palliative chemotherapy rate was 70.0%. Both technical and clinical success rates achieved 100% in our cohort.

### Stent patency

**Figure 2:**
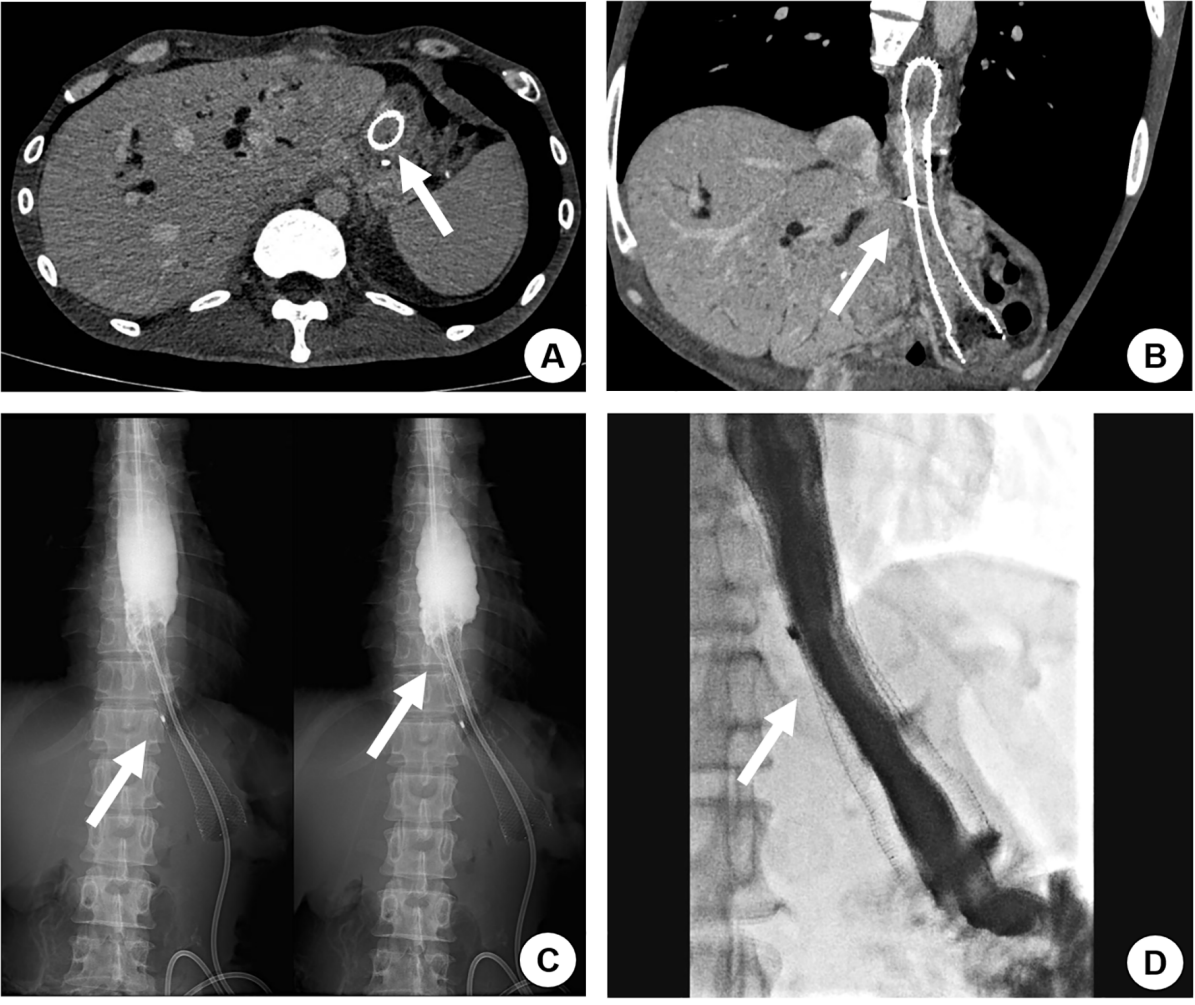
A 42-year-old male with a history of total gastrectomy performed over 1 year prior presented with recurrent nausea and vomiting 131 days after initial self-expanding metal stent (SEMS) placement for esophageal-jejunal anastomotic tumor recurrence with obstruction. Contrast-enhanced CT revealed tumor ingrowth through the stent mesh, causing complete stent occlusion (**A, B**, arrow). Upper gastrointestinal contrast study confirmed the obstruction, demonstrating no contrast passage beyond the stent (**C**, arrow). A second overlapping SEMS was deployed, achieving restored luminal patency with free contrast flow into the distal small intestine on repeat imaging (**D**, arrow). The patient resumed normal oral intake without further obstructive symptoms.

Three patients developed stent occlusion due to intrastent tumor ingrowth secondary to disease progression after initial anastomotic stent placement more than two months, and underwent secondary stent implantation with successful maintenance of patency postoperatively (**Figure 2**). One patient developed stent obstruction due to food impaction on postoperative day 10, which was managed endoscopically with successful restoration and maintenance of luminal patency. One patient was readmitted 30 days after stent placement due to abdominal distension. A colonic stent was inserted to relieve colonic obstruction because of peritoneal metastases invasion of the splenic flexure of the colon, after which the patient’s digestive tract maintain patent, allowing the resumption of oral intake. The others patients kept stent patency during the follow-up period without requiring further intervention. Thus, the mean duration of stent patency was 78 days (interquartile range: 8–225 days).

### Complications

Postprocedural complications included: One case (10%) of febrile infection within 14 days post-implantation, successfully managed with antibiotic therapy. During the study period, two deaths unrelated to stent placement were recorded in our institution, both attributed to disease progression rather than procedural complications: Day 81: Septic shock (secondary to pre-existing condition); Day 193: Acute cardiopulmonary failure. Six deaths occurred at external medical facilities during follow-up. Despite requesting medical records, the exact etiologies (e.g., disease progression vs. treatment-related complications) remained undocumented in our dataset. Other two patients were lost to follow-up, and their current clinical status remains unknown. No procedure-related complications (migration, hemorrhage, or anastomotic disruption) were observed during follow-up.

## Discussion

In global cancer burden assessments, gastric malignancy consistently ranks within the top tier for both disease occurrence and fatality (1). Approximately 20% of patients with advanced gastric cancer develop gastric outlet obstruction (GOO) (18), which severely compromises their quality of life, subsequent therapeutic interventions and adversely affects long-term survival outcomes (19). For patients with unresectable advanced gastric cancer or those deemed unfit for curative resection, palliative gastrojejunostomy could be performed to alleviate obstructive symptoms and improve quality of life (20). Subsequent antitumor therapy may be initiated following nutritional rehabilitation, ultimately extending overall survival (21). However, in most patients with advanced gastric cancer, prolonged GOO leads to severely compromised nutritional status. Even if palliative gastrojejunostomy is attempted, the prolonged recovery period may significantly delay subsequent antitumor therapy, and such interventions do not necessarily translate into meaningful survival benefits (22).

The application of SEMS in patients with advanced gastric cancer complicated by GOO has become well-established, with proven safety and safety (10). Compared to traditional gastrojejunostomy, SEMS offers a minimally invasive approach via natural orifice deployment, resulting in shorter hospital stays and rapid recovery (23). This approach represents the treatment of choice for elderly patients with significant comorbidities who are poor surgical candidates. Post-stent implantation allows prompt restoration of oral feeding, enhances nutritional rehabilitation, and facilitates earlier administration of systemic anticancer therapy, which collectively contribute to improved survival outcomes (24).

Recent progress in pharmacotherapeutic agents and surgical innovations has led to marked improvements in survival outcomes for gastric cancer patients. However, the incidence of anastomotic tumor recurrence has concurrently risen, primarily manifesting as recurrent nausea, vomiting, and feeding intolerance (1). For such patients, due to high surgical and anesthetic risks, significant procedural trauma, prolonged postoperative recovery, or contraindications for surgery arising from prohibitive technical complexity or extensive tumor burden, current evidence suggests limited applicability of repeated surgical resection in recurrent cases (25). Management typically relies on total parenteral nutrition (TPN) or long-term nasogastric/nasojejunal tube feeding for enteral support, resulting in poor quality of life and diminished overall survival.

Previous studies have reported the application of covered-SEMS in patients with anastomotic tumor recurrence and obstruction following gastric cancer surgery (11, 12, 26–28). These stents demonstrated significant improvement in quality of life with acceptable safety profiles, making them a viable therapeutic option for such advanced-stage patients. Clinical outcomes analysis revealed significant advantages of SEMS over traditional operative: minimally invasive approach, lower perioperative complication rates, shorter hospital stays (typically 1–3 days vs. 7–14 days for surgery), rapid resumption of oral intake (within 24–48 hours post-procedure) and cost-effectiveness (26). Compared to nasoenteric tube placement or jejunostomy: dietary flexibility such as allowing consumption of a full or soft diet (vs. liquid-only nutrition with tubes), eliminates physical discomfort and psychosocial burden of external feeding devices; Preserves normal eating behaviors, enhancing patient dignity and social functioning; Reduces long-term complications (e.g., tube dislodgement, skin irritation at stoma sites) (27). In this study, all 10 patients with anastomotic tumor recurrence and obstruction following gastric cancer surgery underwent uncovered SEMS placement at the stenotic anastomotic site. Successful stent deployment and subsequent symptom relief were obtained in every case, with clinically meaningful advancement in dietary tolerance.

Prior studies have demonstrated that covered stents were associated with a higher rate of stent migration compared to uncovered stents, while exhibiting a lower risk of stent occlusion (29–31). Compared to stent obstruction caused by tumor progression, stent migration into the distal small intestine poses a greater clinical risk. If the migrated stent occluded the distal bowel, surgical intervention was necessary. However, such patients were often unable to tolerate additional surgery, resulting in a life-threatening scenario. So, in this study, all patients were treated with an uncovered stent. Stent occlusion secondary to tissue ingrowth represented the predominant adverse event (30%, 3/10 cases). The observed higher occlusion frequency with U-SEMS likely reflects increased susceptibility to tumor proliferation and hyperplastic tissue response through the unconstrained stent interstices. In Patients 2, 3, and 8, tumor progression beyond 2 months post-stent implantation led to stent obstruction via tumor ingrowth through the stent mesh. Salvage therapy employing overlapping stent deployment successfully reestablished luminal patency, enabling resumption of unrestricted oral nutrition. The secondary intervention demonstrated durable efficacy, with no recurrent obstructions observed during longitudinal surveillance.

In this study, all patients were in the advanced stage of malignant tumor, without indication of surgical resection, completely unable to take liquid oral, and kept alive by intravenous fluids. Following SEMS implantation, all patients resumed liquid nutrition within 72 hours. This rapid dietary rehabilitation significantly enhanced both nutritional parameters and quality-of-life metrics. Comparative analysis revealed SEMS enabled earlier oral intake than surgical gastrojejunostomy, supporting its preferential use for obstructive anastomotic recurrences (32). Another exciting thing about this study, after sent placement, 70.0% (7/10) of patients recovered well and continued the next course of chemotherapy or tried a new chemotherapy regimen. The stent patency and overall survival of the patients who received chemotherapy after stent implantation were higher than those who did not (33, 34).

This study has several limitations. Firstly, as a single centre retrospective case series with a small sample size, the findings on stent safety and clinical safety are preliminary and lack high-level evidence-based validation. Secondly, the technical and clinical success rate in this study is extremely high and these results may result from retrospective study selection bias. Thirdly, all included patients had advanced-stage malignancies, and incomplete data due to irregular follow-up precluded analysis of overall survival rates. Lastly, the limited case volume of anastomotic tumor recurrence with obstruction post-gastrectomy prevented comparative evaluation between covered and uncovered stents in this specific population. Future multicentre prospective studies with standardized follow-up protocols are needed to validate the role of SEMS in this clinical scenario.

## Conclusions

Fluoroscopically-guided SEMS placement represents a technically safe and clinically effective intervention for managing anastomotic obstructions in recurrent gastric cancer. SEMS placement offers rapid symptom relief, shorter hospital stays, and improved quality of life compared to surgical alternatives in this patient population. Thus, based on its technical feasibility and clinical outcomes, this method warrants primary consideration in palliative treatment algorithms.

## Data Availability

The original contributions presented in the study are included in the article/supplementary material. Further inquiries can be directed to the corresponding author.
